# The efficacy and safety of acupuncture combined with language training for motor aphasia after stroke: study protocol for a multicenter randomized sham-controlled trial

**DOI:** 10.1186/s13063-022-06280-2

**Published:** 2022-06-30

**Authors:** Shizhe Deng, Bomo Sang, Boxuan Li, Hai Lu, Lili Zhang, Guang Tian, Ting Hao, Yufeng Zhang, Lei Shi, Kaihang Sun, Te Ba, Feng Li, Ying Kong, Mengni Qin, Jianli Zhang, Xiaofeng Zhao, Zhihong Meng

**Affiliations:** 1 First Teaching Hospital of Tianjin University of Traditional Chinese Medicine, Tianjin, China; 2 National Clinical Research Center for Chinese Medicine Acupuncture and Moxibustion, Tianjin, China; 3 Tianjin University of Traditional Chinese Medicine, Tianjin, China

**Keywords:** Motor aphasia, Acupuncture, Sham acupoints, Language training, RCT, WAB, CFCP, Study protocol

## Abstract

**Background:**

Motor aphasia after stroke is a common and intractable complication of stroke. Acupuncture and language training may be an alternative and effective approach. However, the efficacy of acupuncture and language training for motor aphasia after stroke has not been confirmed. The main objectives of this trial are to evaluate the effectiveness and safety of acupuncture and low-intensity, low-dose language training in treating ischemic motor aphasia after stroke from 15 to 90 days.

**Methods:**

This is a multicenter randomized sham-controlled clinical trial. We will allocate 252 subjects aged between 45 and 75 years diagnosed with motor aphasia after stroke with an onset time ranging from 15 to 90 days into two groups randomly in a 1:1 ratio. Patients in the experimental group will be treated with “Xing-Nao Kai-Qiao” acupuncture therapy plus language training, and those in the control group will be treated with sham-acupoint (1 cun next to the acupoints) acupuncture therapy plus language training. All the patients will be given acupuncture and language training for 6 weeks, with a follow-up evaluation 6 weeks after the end of the treatment and 6 months after the onset time. The patients will mainly be evaluated using the Western Aphasia Battery and Chinese Functional Communication Profile, and the incidence of treatment-related adverse events at the 2nd, 4th, and 6th weeks of treatment will be recorded. The baseline characteristics of the patients will be summarized by group, the chi-squared test will be used to compare categorical variables, and repeated measures of analysis of variance or a linear mixed model will be applied to analyze the changes measured at different time points.

**Discussion:**

The present study is designed to investigate the effectiveness and safety of traditional acupuncture therapy and language training in ischemic motor aphasia after stroke and explore the correlation between the treatment time and clinical effect of acupuncture. We hope our results will help doctors understand and utilize acupuncture combined with language training.

**Trial registration:**

ChiCTR ChiCTR1900026740. Registered on 20 October 2019

**Supplementary Information:**

The online version contains supplementary material available at 10.1186/s13063-022-06280-2.

## Administrative information

Note: The numbers in curly brackets in this protocol refer to SPIRIT checklist item numbers. The order of the items has been modified to group similar items (see http://www.equator-network.org/reporting-guidelines/spirit-2013-statement-defining-standard-protocol-items-for-clinical-trials/).

**Table:** 

Title {1}	The efficacy and safety of acupuncture combined with language training for Motor Aphasia after stroke: study protocol for a multicenter randomized sham-controlled trial
Trial registration {2a and 2b}.	Chinese Clinical Trial Registry: ChiCTR1900026740 Registry Name: A randomized controlled trial for “Xing-Nao Kai-Qiao” Rehabilitation Program in the Treatment of Motor Aphasia after Stroke Item 2b: An additional movie file shows this in more detail [see Table 1]
Protocol version {3}	Date: 20 October 2019 Version identifier: V1.1
Funding {4}	This study was funded by the National Key Research and Development Program of China [No. 2018YFC1706001].
Author details {5a}	Shizhe Deng [First Teaching Hospital of Tianjin University of Traditional Chinese Medicine, Tianjin, China; National Clinical Research Center for Chinese Medicine Acupuncture and Moxibustion, Tianjin, China] Bomo Sang [First Teaching Hospital of Tianjin University of Traditional Chinese Medicine, Tianjin, China; National Clinical Research Center for Chinese Medicine Acupuncture and Moxibustion, Tianjin, China] Boxuan Li [First Teaching Hospital of Tianjin University of Traditional Chinese Medicine, Tianjin, China; National Clinical Research Center for Chinese Medicine Acupuncture and Moxibustion, Tianjin, China] Hai Lu [Tianjin University of Traditional Chinese Medicine, Tianjin, China] Lili Zhang [First Teaching Hospital of Tianjin University of Traditional Chinese Medicine, Tianjin, China; National Clinical Research Center for Chinese Medicine Acupuncture and Moxibustion, Tianjin, China] Guang Tian [First Teaching Hospital of Tianjin University of Traditional Chinese Medicine, Tianjin, China; National Clinical Research Center for Chinese Medicine Acupuncture and Moxibustion, Tianjin, China] Ting Hao [Tianjin University of Traditional Chinese Medicine, Tianjin, China]Yufeng Zhang [Tianjin University of Traditional Chinese Medicine, Tianjin, China] Lei Shi [First Teaching Hospital of Tianjin University of Traditional Chinese Medicine, Tianjin, China; National Clinical Research Center for Chinese Medicine Acupuncture and Moxibustion, Tianjin, China] Kaihang Sun [Tianjin University of Traditional Chinese Medicine, Tianjin, China] Te Ba [Tianjin University of Traditional Chinese Medicine, Tianjin, China] Feng Li [Tianjin University of Traditional Chinese Medicine, Tianjin, China] Ying Kong [Tianjin University of Traditional Chinese Medicine, Tianjin, China] Mengni Qin [Tianjin University of Traditional Chinese Medicine, Tianjin, China] Janli Zhang [First Teaching Hospital of Tianjin University of Traditional Chinese Medicine, Tianjin, China; National Clinical Research Center for Chinese Medicine Acupuncture and Moxibustion, Tianjin, China] Xiaofeng Zhao [First Teaching Hospital of Tianjin University of Traditional Chinese Medicine, Tianjin, China; National Clinical Research Center for Chinese Medicine Acupuncture and Moxibustion, Tianjin, China] Zhihong Meng [First Teaching Hospital of Tianjin University of Traditional Chinese Medicine, Tianjin, China; National Clinical Research Center for Chinese Medicine Acupuncture and Moxibustion, Tianjin, China]
Name and contact information for the trial sponsor {5b}	Special funds from the central government of China
Role of sponsor {5c}	The sponsor provides funding to support the design of the study, execution, analysis, interpretation of the data, and decision to submit the results.

**Table 1:** Trial registration data

Data category	Information
Registration number	ChiCTR1900026740
Date of last refreshed on	October 22, 2019
Date of registration	October 20, 2019
Registration status	Prospective registration
Public title	A randomized controlled trial for “Xing-Nao Kai-Qiao” Rehabilitation Program in the Treatment of Motor Aphasia after Stroke
Scientific title	A randomized controlled trial for “Xing-Nao Kai-Qiao” Rehabilitation Program in the Treatment of Motor Aphasia after Stroke
The registration number of the Partner Registry	AMCTR-IOR-19000302
Study leader	Meng Zhihong
Study leader’s address	314 Wes Anshant Road, Nankai District, Tianjin, China
Approved no. of the ethics committee	TYLL2019[K]word015
Primary sponsor	The First Affiliated Hospital of Tianjin University of Traditional Chinese Medicine
Source(s) of funding	Central government special funds
Target disease	Aphasia after stroke
Study type	Interventional study
Study design	Parallel
Inclusion criteria	(1) Meet the diagnostic criteria for motor aphasia after stroke; (2) aphasia appears for the first time after stroke; (3) onset time ranged from 15 days to 3 months; (4) the severity of aphasia is 0–3, which can be used in conjunction with speech training; (5) consciousness of consciousness and stable vital signs; (6) aged 45 to 75 years old; (7) patient or family signed an informed consent form and have a good compliance
Exclusion criteria	(1) Combined with severe primary diseases such as liver, kidney, hematopoietic system, and endocrine system; (2) there are abnormalities in audio-visual, severe cognitive impairment, and mental illness, which cannot be combined with examination and treatment; (3) pregnant and lactating women
Recruiting time	From October 21, 2019, to December 31, 2020
Countries of recruitment	China
Primary outcomes	Western Aphasia Battery; Functional Language Communication Capability Check
Secondary outcomes	Boston Diagnostic Aphasia Examination; NIH Stroke Scale; SAQOL-39 Quality of Life Scale; Stroke-Specific Quality of Life Scale; the Health Scale of Traditional Chinese Medicine
Randomization procedure	Stratified block randomization method
Blinding	Open label
The time of sharing IPD	Within 6 months after the trial complete
The way of sharing IPD	Apply to the main researcher for publication

## Introduction

### Background and rationale {6a}

#### Justification for undertaking the trial

Motor aphasia after stroke (MAAS) is one of the critical complications associated with ischemic stroke because it leads to harmful effects in patients and a significant economic burden to society [1]. Aphasia was reported in approximately 21 to 38% of acute ischemic stroke patients [1–3].

As a Traditional Chinese Medicine (TCM) therapy, acupuncture has been utilized in China for thousands of years. It is effective in treating MAAS to improve functional communication and language function [4]. Additionally, acupuncture has demonstrated positive changes in motor speech symptoms or “motor” aphasia [5]. Some studies have revealed that acupuncture has therapeutic advantages in spontaneous speech, repetition, naming, and daily communication for MAAS patients [6, 7]. Acupuncture may be effective in motor aphasia, but methodological weaknesses in many studies compromise firm conclusions about its efficacy. Thus, the effectiveness of acupuncture must be confirmed in rigorously designed studies with solid evidence from more trial data [8–10].

#### Summary of relevant studies

Presently, transcranial direct current stimulation (tDCS), non-invasive brain stimulation (NIBS), novel speech-language therapy (SLT), pharmacological treatments, and some alternative treatments have been reported to treat motor aphasia after stroke [11]. Although some positive effects have been achieved, some limitations have also been reported in clinical applications. For example, pharmacological treatments might improve the recovery from aphasia after stroke. However, no drug has been proven to produce an overall favorable effect [12]. Language training improves oral naming accuracy for trained items in patients with aphasia, with long-term gain maintenance over time [13]. Thus, SLT may be beneficial at high intensity, high dose, or over a longer period; however, it is not suitable for all patients [14]. Additionally, tDCS and NIBS lack sufficient evidence to improve language function [14, 15].

#### Research question

The main objectives of this trial are to evaluate the efficacy and safety of acupuncture and low-intensity, low-dose language training in ischemic motor aphasia after stroke from 15 to 90 days.

### Objectives {7}

Research hypothesis: “Xing-Nao Kai-Qiao” acupuncture combined with language training is an effective approach for MAAS, and its efficacy is superior to sham-acupoint acupuncture and language training.

Study objectives: To assess the efficacy and safety of “Xing-Nao Kai-Qiao” acupuncture combined with language training on ischemic MAAS from 15 to 90 days.

### Trial design {8}

The clinical study is designed as a multicenter randomized sham-controlled clinical trial. A total of 252 participants will be randomized into two parallel groups at a 1:1 ratio, including the acupuncture group and the sham acupuncture group. Each eligible subject will undergo a 6-week treatment period, followed by a follow-up period of 6 weeks after treatment and 6 months after onset time. Six visits in total are scheduled for each subject at weeks 0, 2, 4, and 6; 6 weeks after treatment; and 6 months after onset time (Table 2).

**Table 2:** Trial process chart

Period		Screening	Treatment			Follow-up	
Items		Before enrolment (weeks) −2 to −1	End of 2-week treatment (weeks) 3	End of 4-week treatment (weeks) 5	End of 6-week treatment (weeks) 7	End of 6 weeks after the end of the treatment follow-up (weeks) 13	End of 6 months after the onset time follow-up
Recruitment		X					
Enrolment		X					
Inclusion criteria		X					
Exclusion criteria		X					
Basic characteristic variables		X					
Randomization and allocation concealment		X					
Primary outcomes	Western Aphasia Battery	X	X	X	X	X	X
	Chinese Functional Communication Profile	X	X	X	X	X	X
Secondary outcomes	X	X	X	X		X	X
	National Institute of Health Stroke Scale	X	X	X	X	X	X
	Stroke and Aphasia Quality of Life Scale-39	X	X	X	X	X	X
	Stroke Specific Quality of Life Scale	X	X	X	X	X	X
	Health Scale of Traditional Chinese Medicine	X	X	X	X	X	X
Adverse events recorded			X	X	X		
Relapse						X	X
Self-reported drug therapy			X	X	X	X	X
Compliance			X	X	X		

## Methods: participants, interventions, and outcomes

### Study setting {9}

The patient data will be collected throughout the clinical trial in China at three level-III hospitals in Tianjin, Changchun, and Jinan.

### Eligibility criteria {10}

Inclusion criteria:

1. Subjects diagnosed with ischemic stroke according to the International Classification of Diseases code ICD-10-I63.902 [16]
2. Subjects diagnosed with aphasia for the first time after stroke
3. Subjects diagnosed with aphasia ranged from 15 to 90 days
4. Severity of aphasia of 0–3 can be combined with speech training
5. Awake consciousness and stable vital signs
6. Subjects aged between 45 and 75 years, male or female
7. Subjects and their families fully understand the research content and agree to participate in the study and sign the informed consent form

Exclusion criteria:

1. Subjects diagnosed with aphasia not caused by stroke
2. Subjects with aphasia before the onset time
3. Subjects with severe heart disease and kidney and liver function insufficiency
4. Subjects with audio-visual abnormalities, severe cognitive impairment, and mental illness cannot cooperate with examination and treatment
5. Pregnant or lactating women

Dropout criteria:

1. Subjects have poor compliance during the clinical study and are unwilling to accept the intervention and withdraw voluntarily
2. Subjects experience severe adverse reactions or deterioration during the clinical study and are not eligible for further intervention

### Who will take informed consent? {26a}

Before the start of clinical studies, trained researchers will introduce the trial to patients and discuss the trial with patients considering the information informed, including the research nature, research purpose, possible benefits and dangers, meeting the requirements of the Declaration of Helsinki, and the rights and obligations of the subjects. Patients will be able to have informed discussions with researchers. The subjects or their legal representative will understand, agree, and sign the written informed consent. Researchers will obtain written consent from patients willing to participate in the trial.

### Additional consent provisions for collection and use of participant data and biological specimens {26b}

Not applicable. We have not designed ancillary studies.

## Interventions

### Explanation for the choice of comparators {6b}

Acupuncture therapy and language training are recommended as a control group. Manipulators should choose acupoints that are 1 cun away from the acupoints of the verum acupuncture group. The stimulating amount of acupuncture requires no “De Qi” sensation. Sham-acupoint acupuncture mainly consists of some sham acupoints not located on the meridians and have no therapeutic effect on aphasia.

### Intervention description {11a}

Subjects in the experimental group will receive verum acupuncture and language training, and those in the control group will receive sham-acupoint acupuncture and language training. Additionally, common drugs used in the neurological department are allowed, such as anti-hypertensive drugs and antiplatelet drugs, which are included in the Guidelines for the Diagnosis and Treatment of Integrated Traditional Chinese and Western Medicine in Cerebral Infarction in China (2017). The same individuals will deliver the trial interventions in all the study groups.

#### Experimental group

##### Verum acupuncture

Disposable sterile acupuncture needles (0.25 mm × 40 mm; Hwato brand; Suzhou Medical Supplies Factory Co. LTD, Suzhou, China) were used. “Xing-Nao Kai-Qiao” acupuncture is applied to treat MAAS according to TCM theory. The acupoints include bilateral PC6 (Neiguan), GV26 (Shuigou), affected side SP6 (Sanyinjiao), and CV23 (Lianquan), beside CV23 (left Panglianquan and right Panglianquan), and affected side HT1 (Jiquan), affected side LU5 (Chize), and affected side BL40 (Weizhong).

Acupoints PC6 and BL40 are inserted at a depth of 5–10 mm, acupoint CV23 (left Panglianquan and right Panglianquan) is inserted at a depth of 55 mm, acupoint GV26 is inserted at a depth of 5–10 mm, and the others are inserted at a depth of 25–35 mm. The pecking technique is performed on GV26 to achieve an intense 6–9-s stimulation. In the other group, lifting-pushing and spinning-rotating methods were performed to achieve the “De Qi” sensation, such as pain, numbness, heaviness, swelling, etc. Finally, after 30 min of retention, all needles are removed. Participants will receive treatment for 6 consecutive weeks, 5 times a week.

The manipulators are senior professional doctors with profound background knowledge and rich experience accumulated for many years.

#### Control group

##### Language training

Some rehabilitation language training is allowed, such as listening comprehension therapy training and reading comprehension therapy training, which are included in the Operational Specifications for Commonly Used Rehabilitation Techniques (2012 edition). The treatment duration is 60 min each at 5 sessions per week for 6 consecutive weeks.

The procedure is performed by exceptional language therapists who have been professionally trained.

##### Sham-acupoint acupuncture

Sham-acupoint acupuncture mainly consists of some sham acupoints not located on the meridians and have no therapeutic effect on aphasia. According to the WTO international standards of acupoint selection, we select the acupoints to be studied. Also, with the acupoints as a reference, the lateral opening of 1 cun in the horizontal direction selected non-meridian and non-acupoint as a control study. The stimulating amount of acupuncture requires no “De Qi” sensation. The same procedure is used as that for the verum acupuncture group.

All the members who participated in the study will get a series of formal training on the study protocol, treatment methods, and assessment. All acupuncturists and speech therapists will receive training related to the study prior to recruitment, including the location of acupuncture points, depth, direction of stabbing, frequency of lifting and inserting, and speech training techniques.

### Criteria for discontinuing or modifying allocated interventions {11b}

Improved health status: exacerbations, complications, abnormal laboratory results, and adverse events of death from all causes during the observation period.

Withdrawal of participant consent: not being able to visit the clinic for continuous treatment due to inconvenient transportation or a lack of escorts.

### Strategies to improve adherence to interventions {11c}

Through scientifically informed consent, active health education, strengthening humanized care, and making long-term follow-up plans, the subject’s compliance will be improved. We created record cards that included the date of treatment, personal information, and the therapist’s signature to document patient compliance. The participants are also subsidized for transportation expenses.

### Relevant concomitant care permitted or prohibited during the trial {11d}

The patients are prohibited from receiving treatments such as tDCS, NIBS, additional SLT, and some alternative therapies during the intervention period.

### Provisions for post-trial care {30}

Adverse reactions associated with needling include subcutaneous bleeding or hematoma and pain around the needling point. The investigator shall conduct a corresponding examination and symptomatic treatment for the expected adverse effects. Researchers shall take necessary treatment measures according to the condition for possible unexpected adverse events until the adverse events disappear. According to the severity of adverse events, we can choose inpatient, outpatient, home visit, telephone, Internet-based, and other treatment modes.

### Outcomes {12}

#### Primary outcomes

The main observation was the difference between the two groups in WAB [17] scale scores and CFCP [18] scale scores after 6 weeks of treatment to assess the patients’ language abilities and functional communication abilities.

#### Secondary outcomes

The secondary outcome measures were WAB scale scores and CFCP scale scores, assessing the difference between the two groups at 6 months after onset time; Boston Diagnostic Aphasia Examination (BDAE) [19] scores for the severity of the language competence status; Stroke and Aphasia Quality of Life Scale-39 (SAQOL-39) [20] and Stroke-Specific Quality of Life Scale (SS-QOL) [21] scores for the quality of living in stroke patients; National Institute of Health Stroke Scale (NIHSS) [22] scores for the neurological deficit; Health Scale of Traditional Chinese Medicine (HSTCM) [23] scores for the health status, assessing the difference between the two groups after 6 weeks of treatment and 6 months after onset time. The study’s adverse events and dropout causes will be recorded in a case report form (CRF).

### Participant timeline {13}

**Fig. 1:**
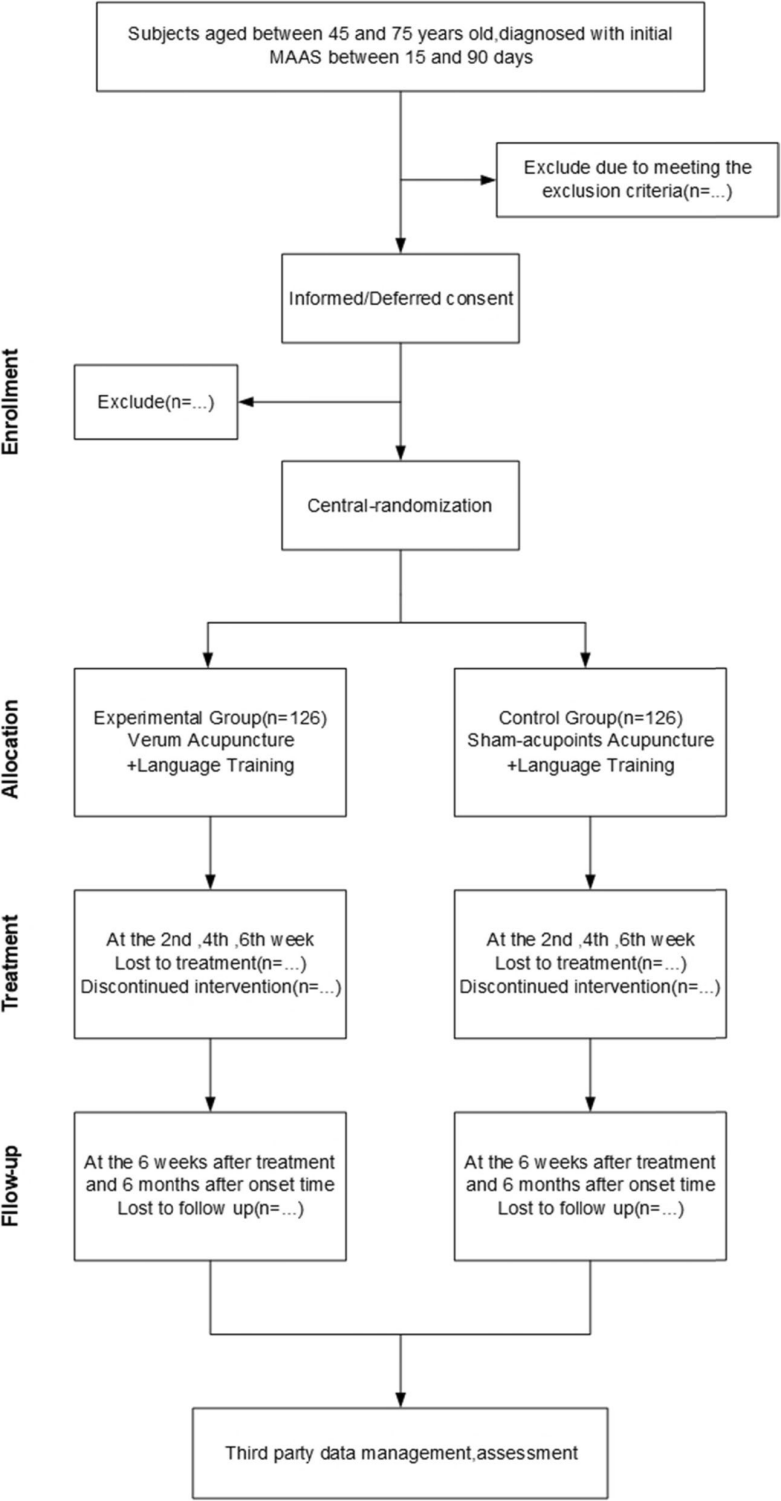
Study design and participation flow chart

The participant timeline is shown in Fig. 1.

### Sample size {14}

Based on the previous pilot study, we expected that acupuncture combined with speech rehabilitation would be more effective than placebo combined with speech rehabilitation [6]. We were assuming that the difference in WAB-AQ values after treatment between acupuncture and placebo is 10.9, with a standard deviation of 22.9 in both groups. According to expert clinical observation, it was found that a superiority margin of 3 for comparisons of acupuncture and placebo for the primary outcome. We performed a superiority test to calculate the appropriate trial sample size with 80% power, *β* =0.2, and *α* = 0.05. The results showed that a clinically significant difference could be detected by a sample size of at least 105 in each group, allowing for a predicted 20% dropout rate. We plan to enroll 252 participants in this study.

### Recruitment {15}

The study will be conducted at the following three hospitals: First Teaching Hospital of Tianjin University of Traditional Chinese Medicine, Changchun University of Chinese Medicine, and QILU Hospital of Shandong University. Participants diagnosed with ischemic stroke with aphasia will be recruited at three hospitals, mainly from clinical processes. Also, we will recruit subjects through posters and flyers. The subject screening will be completed at each center by experienced researchers. Informed consent of eligible patients was obtained and then randomized into groups.

## Assignment of interventions: allocation

### Sequence generation {16a}

The China Academy of Chinese Medical Sciences was commissioned to apply the “Central Randomization System for Clinical Research.” The 252 subjects will be randomly assigned to the experimental group and control group at 1:1 using the district-group randomization method.

### Concealment mechanism {16b}

The participants will be randomized using the “Central Randomization System for Clinical Research.” The central randomization system was established to implement the allocation sequence and conceal the sequence until interventions are assigned. Throughout the study, Central will perform randomization and maintain the list, continuously blinded to data management and statisticians until the database is closed.

### Implementation {16c}

All subjects who meet the inclusion criteria and sign the informed consent form will be randomized. Requirements for randomization will be the responsibility of the investigator responsible for recruitment. The overall research group assigns the username and password to the quality control personnel in each sub-center. The quality control personnel logs into the central random system according to the respective username and password and applies for the random number online.

## Assignment of interventions: blinding

### Who will be blinded {17a}

Trial participants, outcome assessors, and statistical analysts will be blinded after being assigned to the intervention. Trial participants are randomly assigned. The outcome assessor enters the measurements into the computer, and the statistical analyst analyzes the data without access to the assignment information. The data will be reviewed in a blinded manner, entrusting the third-party (Guangdong Province Traditional Chinese Medical Hospital) medical statistics professionals to conduct statistical analysis.

### Procedure for unblinding if needed {17b}

Not applicable. Unblinding is not performed until the study is over.

## Data collection and management

### Plans for assessment and collection of outcomes {18a}

The original data include the patient’s baseline and medical documents of the subject, including outpatient medical records, inpatient medical records, and physical and chemical examination reports. Clinical research process documents include the informed consent form, screening form, inclusion form, medication record, laboratory record, and research case report form.

The scales used such as WAB, CFCP, BDAE, SAQOL-39 and SS-QOL, NIHSS, and HSTCM are all approved by the domestic and international industries. Two standardized trained physicians with the same criteria in each sub-center will perform the scale assessment to reduce variation between centers.

The quality supervisor will regularly verify the test process and check the authenticity of the data to ensure the quality of the test. Meanwhile, the data will be independently managed by a third party.

### Plans to promote participant retention and complete follow-up {18b}

The doctors in charge of the hospital follow up the patients by outpatient re-examination 6 weeks after the end of the treatment and 6 months after the onset time, performing scale measurement and medication evaluation, and collecting and storing the follow-up information of the patients. To reduce the rate of loss to follow-up, we will contact the patient or his/her family according to the follow-up date, make an appointment for the follow-up time, and give the patient a transportation subsidy of 100 yuan per person for each follow-up. If no contact can be made by telephone or at home, a loss to follow-up will be recorded. The causes of case dropout, discontinuation, and exclusion during the clinical trial shall be understood and documented in detail. The time of the last treatment will be recorded, and the items that can be evaluated will be completed.

### Data management {19}

All the data will be recorded in double versions using computer software, and different inputters will be trained. They will input the same case report and check with each other, and the two versions will be stored in the database.

Data collection shall be timely, complete, and accurate. After a blind audit of the data is conducted and the database established is deemed correct, the primary researcher and statistical analyst will lock the data. Researchers shall keep all study data until 5 years after the end of the study.

### Confidentiality {27}

All information related to the study will be securely stored at the study site. Information about all participants will be held in a restricted area. All data collection, processing, and management firms will be coded and classified to keep subject information confidential. All records containing names or other personal information, such as informed consent, will be kept separate from study records. This information will be kept in a specific area.

### Plans for collection, laboratory evaluation, and storage of biological specimens for genetic or molecular analysis in this trial/future use {33}

Not applicable. We will not design or collect biological specimens for genetic or molecular analysis in the current trial.

## Statistical methods

### Statistical methods for primary and secondary outcomes {20a}

Baseline characteristics of patients were summarized in groups according to the study data. The chi-square test was used to compare categorical variables, and the 2-sample *t*-test or Wilcoxon rank-sum test was used to compare continuous variables. Analysis of variance for repeated measures was applied according to the outcome of the measurement time, or linear mixed models were used to analyze changes in measurements at different time points. Covariates such as age, duration of illness, and severity of aphasia will be included in the statistical model, correcting the effects of possible imbalances. If there is a violation of the distribution assumption, the appropriate transformation will be used.

### Interim analyses {21b}

Not applicable because we will not perform an interim analysis.

### Methods for additional analyses (e.g., subgroup analyses) {20b}

Not applicable because we will not perform additional analyses.

### Methods in analysis to handle protocol non-adherence and any statistical methods to handle missing data {20c}

Data may be missing. Therefore, we will analyze data based on intention-to-treat analysis (ITT) and per-protocol analysis (PP) principles. We will also assess the robustness of the results of our research by comparing the results of the two datasets mentioned above to evaluate group effects.

### Plans to give access to the full protocol, participant-level data, and statistical code {31c}

The release of raw data is 6 months after completing the trial following a request to the principal investigator.

## Oversight and monitoring

### Composition of the coordinating center and trial steering committee {5d}

The principal investigator and study physicians will design and conduct and prepare protocols, investigator manuals, and case report forms (CRFs). In addition, they will organize steering committee meetings, manage the clinical trial office, and publicize study reports. They are members of the trial management committee. The steering committee will consist of a manager from the project contractor, and each sub-center will be responsible for recruiting subjects, advancing the study’s progress, and ensuring its successful completion. The study team will be responsible for subject recruitment, collection of trial data, filling CRF, and completing subject follow-up according to the study protocol and investigator manual. The data manager is responsible for data entry and validation.

#### Public or patient participation

In recent times, there has been a paradigm shift towards patient-centered care and active involvement of patients and the public in research design and active participation throughout the research process. However, no public or patient involves in the design of our program.

### Composition of the data monitoring committee, its role, and reporting structure {21a}

The data supervision committee comprises a four-level quality inspection system. Each clinical research unit appoints a supervisor. The clinical lead unit and project undertaking unit respectively send a supervisor to set up a supervision team to monitor the data collected by the clinic, ensuring the authenticity and reliability of the project. This committee has no conflict of interest with the funders.

### Adverse event reporting and harms {22}

Subcutaneous bleeding or hematoma around acupoints during acupuncture therapy and pain around acupoints related to acupuncture are expected adverse reactions and do not need to be reported to the ethics committee. Only unexpected serious adverse events, such as deterioration and death from all causes throughout the treatment period, should be reported immediately to the principal investigator and further to the ethics committee. All details will be recorded, and appropriate medical attention will be given immediately.

### Frequency and plans for auditing trial conduct {23}

This study will conduct an interim trial review, independent of the investigator and sponsor.

### Plans for communicating important protocol amendments to relevant parties (e.g., trial participants, ethical committees) {25}

Any possible modification to the protocol during the trial, including changes in study design, sample size, or study procedures, will require a formal modification to the protocol. The modification will be agreed upon by the project study team, approved by the ethics committee prior to implementation, and notified to the Chinese government’s Ministry of Science and Technology.

### Dissemination plans {31a}

Trial results will be published in the Clinical Trials Registry database or made available to medical professionals and the public through publications upon completion of the entire clinical trial (Table 1).

## Discussion

Acupuncture has been increasingly used to treat motor aphasia after stroke in the past two decades [24]. A study found that acupuncture combined with language training could improve the early rehabilitation effect of MAAS [25]. Other studies have demonstrated that specific acupoint stimulation activated the language-related areas in the brain of MAAS patients [26–28].

Although acupuncture has therapeutic effects on aphasia after stroke [24], it has not been recommended in the current stroke guidelines because of methodology limitations in relevant studies [29]. The efficacy of acupuncture requires methodologically rigorous, high-quality RCTs to confirm [4, 11, 30].

To distinguish between the acupuncture and placebo effects, we choose needling 1 cun away from the acupoints of the actual acupuncture group as sham acupuncture; these acupoints are not located on the meridians and do not affect aphasia. Due to the physical stimulation of acupuncture and familiarity of Chinese patients with acupuncture, double-blind and non-penetration sham acupuncture could not be performed in this study. Instead, non-acupuncture and non-meridian acupoint shallow acupuncture are used. However, slight physical stimulation will occur in the location of acupoints that will not affect the evaluation of acupuncture efficacy.

The study will adopt WAB as primary outcomes, commonly used to evaluate language function in clinical practice. WAB uses clinical and neurolinguistic principles to assess the severity of the spoken language deficit in aphasia and has high internal consistency, test-retest reliability, and validity [31]. Both CFCP and BDAE can assess the language ability of aphasia patients. BDAE has been demonstrated to have good discriminant validity in different languages and cultural backgrounds [32]. CFCP can better focus on evaluating the changes in daily life communication of aphasia patients and providing language rehabilitation training guidance [33]. Additionally, patients with TCM treatment will evaluate the neurological function, quality of life, and health status using NIHSS, SAQOL-39, SS-QOL, and HSTCM.

In addition, to reduce possible bias and improve study quality, we will follow standardized procedures for randomization and allocation concealment. Researchers, outcome assessors, and data statisticians will be blinded. All procedures, including acupuncture point extraction, manipulation, and efficacy assessment, will be performed in strict accordance with standardized methods.

In conclusion, we expect to demonstrate the efficacy and safety of acupuncture combined with speech training in ischemic MAAS. We hope that the results of this study will help clinicians gain further insight into acupuncture combined with speech training therapy, as well as provide solid evidence for future research on the mechanisms of MAAS.

## Trial status

Registration number: ChiCTR1900026740

The registration number of the Partner Registry or other register: AMCTR-IOR-19000302

Date: 20 October 2019

Version identifier: V1.1

The date recruitment began: 21 October 2019

The date recruitment will be completed: 31 December 2020

## Supplementary Information

**Additional file 1.**

**Additional file 2.**

**Additional file 3.**

**Additional file 4.**

## Abbreviations

MAAS: Motor aphasia after stroke
tDCS: Transcranial direct current stimulation
NIBS: Non-invasive brain stimulation
SLT: Speech and language therapy
AQ: Rating scale
STRICTA: Standards for Reporting Interventions in Clinical Trials of Acupuncture
TCM: Traditional Chinese Medicine
WAB: Western Aphasia Battery
CFCP: Chinese Functional Communication Profile
BDAE: Boston Diagnostic Aphasia Examination
NIHSS: National Institute of Health Stroke Scale
SAQOL-39: Stroke and Aphasia Quality of Life Scale-39
SS-QOL: Stroke Specific Quality of Life Scale
HSTCM: Health Scale of Traditional Chinese Medicine
CRF: Case report form
ITT: Intention-to-treat analysis
PP: Per-protocol analysis
