# Supplementary material for: The efficacy and safety of acupuncture combined with language training for motor aphasia after stroke: study protocol for a multicenter randomized sham-controlled trial
Source: Trials. 2022 Jun 30;23:540. doi: 10.1186/s13063-022-06280-2 (PMC9245218; doi:10.1186/s13063-022-06280-2)
Supplement: Supplementary file 1 — Additional file 1. [file 13063_2022_6280_MOESM1_ESM.docx]

IEC of The First Affiliated Hospital of Tianjin University of Troditional Chinese Medicine

Approval Notice

Ethical approval No. :TYLL2019[K] word 015

According to the ministry of health “the involved person's biomedical research ethics review method "(2016), the state management bureau of traditional Chinese medicine ”Chinese medicine clinical research ethical review management norms "(2010), the state food and drug administration “the drug clinical trial ethics review guidelines" (2010), the quality control standard for clinical trials (2003), and the world medical association declaration of Helsinki (2013), the international organization for medical science council” the human body biomedical research international ethical guidelines "(2016) principles of ethics, after the review of the Medical Ethics Committee of the First Affiliated Hospital of Tianjin University of Chinese Medicine on August 27, 2019,it was agreed to carry out clinical research on the evidence-based research project of the rehabilitation program of "Xing Nao Kai Qiao" for the treatment of Broca aphasia after stroke, which is jointly applied by the sponsor of the First Affiliated Hospital of Tianjin University of Chinese Medicine and the main postgraduate Meng Zhihong.

Sponsors and researchers are requested to carry out clinical studies in strict accordance with GCP regulations and the protocols approved by this Ethics Committee (version No. : 1st version No. : 20190627) and informed consent (version No. : 1st version No. : 20190627).Clinical trial registration (including registration with the Medical Research Registration Information System) shall be completed before the commencement of the study. If any of the following occurs during the project, a timely written report shall be submitted to the Ethics Committee. Any modification to the informed consent of the clinical protocol; replacement of the principal investigator; occurrence of serious adverse events; occurrence of any situation that may affect the conduct of the trial or increase the risk of subjects; Violation of protocol; suspension or early termination of the clinical study.

The Ethics Committee will conduct a follow-up review of the project at a frequency of 12 months. Please submit a research progress report before July 27, 2020. After completion of the project, please submit the final report.

This approval is valid for August 27, 2019 solstice and August 27, 2022.

IEC of The First Affiliated Hospital of Tianjin University of Troditional Chinese Medicine

Signature of Chairman:Wu Baosheng

Date:2019.08.27

Contacts:Jia Jingyun Contact No.:022-27986258
