# Supplementary material for: The efficacy and safety of acupuncture combined with language training for motor aphasia after stroke: study protocol for a multicenter randomized sham-controlled trial
Source: Trials. 2022 Jun 30;23:540. doi: 10.1186/s13063-022-06280-2 (PMC9245218; doi:10.1186/s13063-022-06280-2)
Supplement: Supplementary file 2 — Additional file 2. [file 13063_2022_6280_MOESM2_ESM.pdf]

天津中医药大学第一附属医院医学伦理委员会
IEC of The First Affiliated Hospital of Tianjin University of Traditional Chinese Medicine

审 查 批 件

Approval Notice

伦理批件号：TYLL2019[K]字 015

根据卫生部《涉及人的生物医学研究伦理审查办法》(2016)、国家中医药管理局《中医药临床研究伦理审查管理规范》(2010)、国家食品药品监督管理局《药物临床试验伦理审查工作指导原则》(2010)、《药物临床试验质量管理规范》(2003)，以及世界医学会《赫尔辛基宣言》(2013)、国际医学科学组织理事会《人体生物医学研究国际伦理指南》(2016)的伦理原则，经天津中医药大学第一附属医院医学伦理委员会 2019 年 8 月 27 日会议审查，同意由申办者天津中医药大学第一附属医院和主要研究者孟智宏共同申请的"醒脑开窍"康复方案治疗中风后运动性失语的循证研究项目开展临床研究工作。

请申办者、研究人员严格遵循 GCP 规定和本伦理委员会批准的方案（版本号：第 1 版 版本日期：20190627）、知情同意书版本号：(版本号：第 1 版 版本日期：20190627）开展临床研究。在研究开始前，须完成临床试验注册（包括医学研究登记备案信息系统登记）。该项目进行中如发生下列情况，须及时书面报告本伦理委员会：①对临床方案、知情同意书等的任何修改；②更换主要研究者；③发生严重不良事件；④出现任何可能影响试验进行或增加受试者危险的情况；⑤出现违反方案情况；⑥暂停或提前终止临床研究。

本伦理委员会将对该项目进行跟踪审查，跟踪审查频率为 12 个月，请于 2020 年 7 月 27 日前提交研究进展报告。项目完成后，请提交结题报告。

本批件有效期为 2019 年 8 月 27 日至 2022 年 8 月 27 日。

天津中医药大学第一附属医院医学伦理委员会

主任委员签字：[illegible]

日　　期：2019.8.27

联系人：贾景蕴　　联系电话：022-27986258

附件 1

天津中医药大学第一附属医院医学伦理委员会

IEC of The First Affiliated Hospital of Tianjin University of Traditional Chinese Medicine

提交审查项目基本情况

Basic Information of The Submitted Project

项目名称	"醒脑开窍"康复方案治疗中风后运动性失语的循证研究		
申办单位	天津中医药大学第一附属医院		
临床研究分类	科研课题	临床研究批文	国家科技部 2018YFC1706001
所属专业	针灸科	主要研究者	孟智宏
研究单位	天津中医药大学第一附属医院、长春中医大学、山东大学齐鲁医院		
审查类型	初始审查	审查方式	会议审查
审查时间	2019 年 8 月 27 日	审查地点	行政楼第三会议室
审阅资料	1、提请伦理审查申请书<br>2、临床研究批文<br>3、主要研究者履历、利益冲突声明、参研人员履历表<br>4、专业科室人员配备及设备设施情况介绍<br>5、研究者手册<br>6、临床试验方案（版本号：第 1 版 版本日期：20190627）<br>7、临床试验方案摘要<br>8、知情同意书（版本号：第 1 版 版本日期：20190627）<br>9、CRF		

试验是否采用盲法？	是（双盲　单盲 √）		否（请在相应处打 √）
研究者资格是否符合要求？	符合 √	不符合	（请在相应处打 √）
人员配备是否符合要求？	符合 √	不符合	（请在相应处打 √）
设备是否符合要求？	符合 √	不符合	（请在相应处打 √）
受试者招聘方法是否恰当？	恰当 √	不恰当	（请在相应处打 √）
向受试者提供的资料是否完整？	完整 √	不完整	（请在相应处打 √）
《知情同意书》是否规范？	规范 √	不规范	（请在相应处打 √）
受试者参加试验有无补偿？	有 √	无	（请在相应处打 √）
发生不良反应或意外时	有及时抢救措施 √	无及时抢救措施（请在相应处打 √）	

联系人：贾景蕴　　联系电话：022-27986258

天津中医药大学第一附属医院医学伦理委员会
IEC of The First Affiliated Hospital of Tianjin University of Traditional Chinese Medicine

表决结果

Decision of The Vote

审 查 日 期： 2019年第四次会议（2019年8月27日）

审查项目名称： "醒脑开窍"康复方案治疗中风后运动性失语的循证研究

主 要 研 究 者： 孟智宏

申 办 单 位： 天津中医药大学第一附属医院

与会委员表决情况：

全体委员13人　　出席10人　　缺席3人

同意	（10）票
作必要修正后同意	（0）票
作必要修正后重审	（0）票
不同意	（0）票
终止或暂停已批准的试验	（0）票

结论：

■同意

□作必要修正后同意

□作必要修正后重审

□不同意

□终止或暂停已批准的试验

主任委员签字： 张云玲

日　　期： 2019.8.27.

联系人：贾景蕴　　联系电话：022-27986258

附件 2

天津中医药大学第一附属医院医学伦理委员会
IEC of The First Affiliated Hospital of Tianjin University of Traditional Chinese Medicine

伦理委员会成员签到表

Signature of IEC Members

临床科研分会

姓　名	职　务	性别	专业情况	单　位	签　字
吴宝新	主任委员	男	医　学	天津中医药大学第一附属医院	[illegible]
张军平	委员	男	中医学	天津中医药大学第一附属医院	[illegible]
王　舒	委员	男	中医学	天津中医药大学第一附属医院	[illegible]
胡思源	委员	男	中医学	天津中医药大学第一附属医院	[illegible]
李　进	委员	男	中药学	退休	
郭从容	委员	女	中医学	天津中医药大学第一附属医院	[illegible]
曹艺龄	委员	女	法　律	北京市兰台律师事务所	[illegible]
刘新桥	委员	男	中医学	天津中医药大学第一附属医院	[illegible]
曹树军	委员	男	管　理	天津中医药大学第一附属医院	[illegible]
刘玉珍	委员	女	中医学	天津中医药大学第一附属医院	[illegible]
李正全	委员	男	管　理	天津中医药大学第一附属医院	
王金贵	委员	男	中医学	天津中医药大学第一附属医院	
贾景蕴	委员	女	护理学	天津中医药大学第一附属医院	[illegible]
