# Supplementary material for: The efficacy and safety of acupuncture combined with language training for motor aphasia after stroke: study protocol for a multicenter randomized sham-controlled trial
Source: Trials. 2022 Jun 30;23:540. doi: 10.1186/s13063-022-06280-2 (PMC9245218; doi:10.1186/s13063-022-06280-2)
Supplement: Supplementary file 3 — Additional file 3. [file 13063_2022_6280_MOESM3_ESM.docx]

Agreement on the organization and implementation of the key special subject of the National Key R&D Program of China "Research on the Modernization of Traditional Chinese Medicine"

Project name: the evidence-based research project of the rehabilitation program of "Xing Nao Kai Qiao" for the treatment of Broca aphasia after stroke

Project undertaker:The First Affiliated Hospital of Tianjin University of Troditional Chinese Medicine

Project leader:Meng Zhihong

Participant: Changchun University of Troditional Chinese Medicine

Person in charge of the project participant: Zheng Peng

Term of execution: December 2018 to December 2021

Party A: The First Affiliated Hospital of Tianjin University of Troditional Chinese Medicine

Party B: Changchun University of Troditional Chinese Medicine

Project entity undertaking The First Affiliated Hospital of Tianjin University of Troditional Chinese Medicine and subject to participate in the unit of Changchun University of Troditional Chinese Medicine, according to the Ministry of Finance and the Ministry of Science and Technology about print and distribute "measures for the administration of the National Key R&D Program of China" of the notification (wealth of science [2016] no. 113) and other relevant documents, as well as the relevant laws, policies and management requirements, the unanimous negotiation between both parties, agreed to "Xing Nao Kai Qiao" rehabilitation of patients with Broca aphasia after stroke evidence-based research topic study reached the following agreement:

Article 2: Allocation of funds

The special funds obtained by applying for this project shall be used strictly in accordance with relevant national regulations to ensure the special funds are used exclusively.

1. According to the division of research tasks and the project assignment, the total special fund for this project is 1.81 million yuan, of which Party B's fund is 223,300 yuan.

2. Party A shall transfer the research funds to Party B in a timely manner upon recept of the special funds issued.

Party A: The First Affiliated Hospital of Tianjin University of Troditional Chinese Medicine

Project leader: Meng Zhihong

Legal representative: Mao Jingyuan

Date of signing: December 11, 2018

Party B: Changchun University of Troditional Chinese Medicine

Project leader: Zheng Peng

Legal representative: Song Bailin

Date of signing: December 11, 2018

Agreement on the organization and implementation of the key special subject of the National Key R&D Program of China "Research on the Modernization of Traditional Chinese Medicine"

Project name: the evidence-based research project of the rehabilitation program of "Xing Nao Kai Qiao" for the treatment of Broca aphasia after stroke

Project undertaker:The First Affiliated Hospital of Tianjin University of Troditional Chinese Medicine

Project leader:Meng Zhihong

Participant: Qilu Hospital of Shandong University

Person in charge of the project participant: Yue Gonglei

Term of execution: December 2018 to December 2021

Party A: The First Affiliated Hospital of Tianjin University of Troditional Chinese Medicine

Party B:Qilu Hospital of Shandong University

Project entity undertaking The First Affiliated Hospital of Tianjin University of Troditional Chinese Medicine and subject to participate in the unit of Qilu Hospital of Shandong University, according to the Ministry of Finance and the Ministry of Science and Technology about print and distribute "measures for the administration of the National Key R&D Program of China" of the notification (wealth of science [2016] no. 113) and other relevant documents, as well as the relevant laws, policies and management requirements, the unanimous negotiation between both parties, agreed to "Xing Nao Kai Qiao" rehabilitation of patients with Broca aphasia after stroke evidence-based research topic study reached the following agreement:

Article 2: Allocation of funds

The special funds obtained by applying for this project shall be used strictly in accordance with relevant national regulations to ensure the special funds are used exclusively.

1.According to the division of research tasks and the project assignment, the total special fund for this project is 1.81 million yuan, of which Party B's fund is 223,300 yuan.

2.Party A shall transfer the research funds to Party B in a timely manner upon recept of the special funds issued.

Party A: The First Affiliated Hospital of Tianjin University of Troditional Chinese Medicine

Project leader: Meng Zhihong

Legal representative: Mao Jingyuan

Date of signing: December 11, 2018

Party B: Qilu Hospital of Shandong University

Project leader: Yue Gonglei

Legal representative: Li Xingang

Date of signing: December 12, 2018
