# Supplementary figures and images for: The efficacy and safety of acupuncture combined with language training for motor aphasia after stroke: study protocol for a multicenter randomized sham-controlled trial
Source: Trials. 2022 Jun 30;23:540. doi: 10.1186/s13063-022-06280-2 (PMC9245218; doi:10.1186/s13063-022-06280-2)

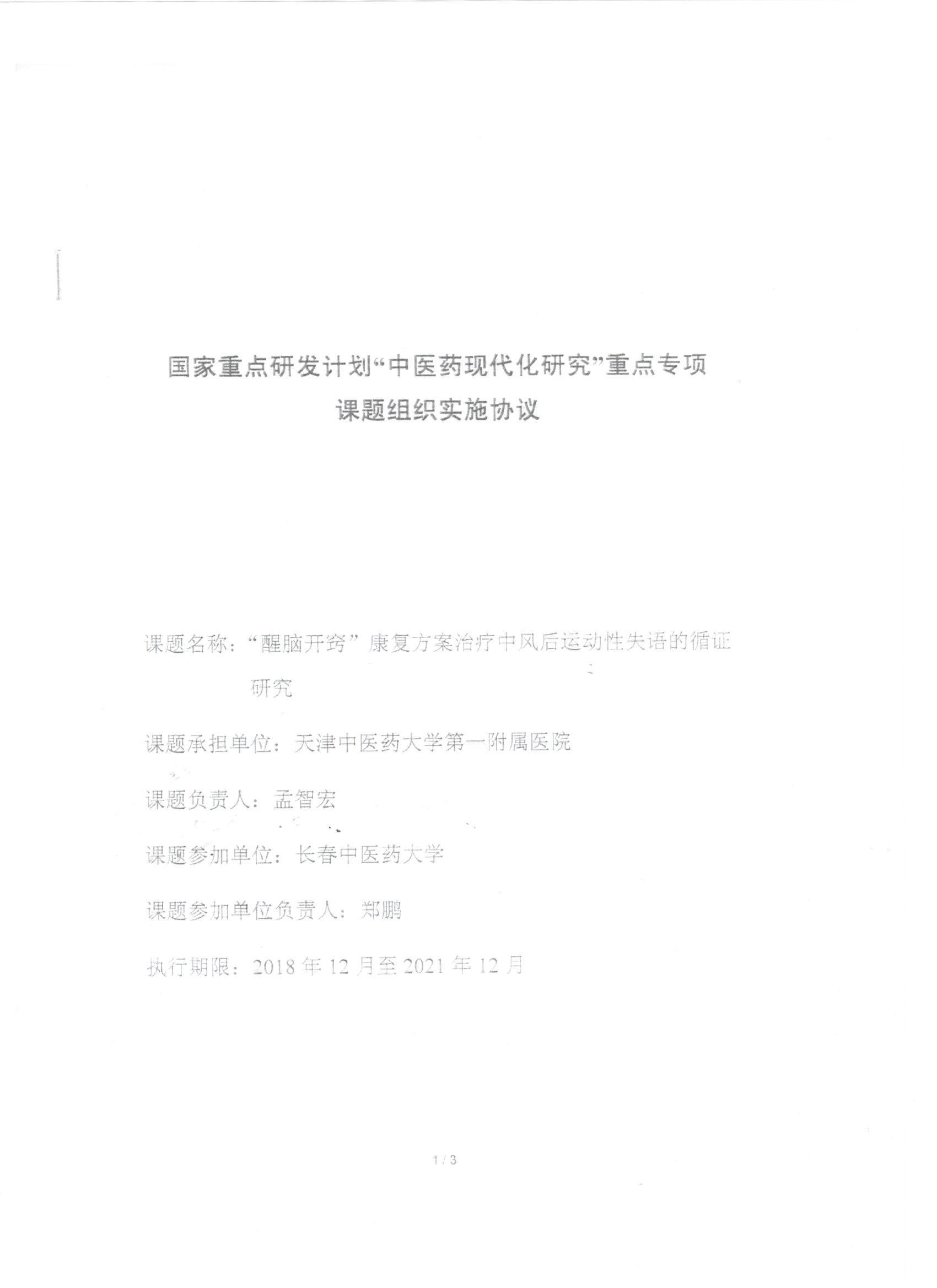


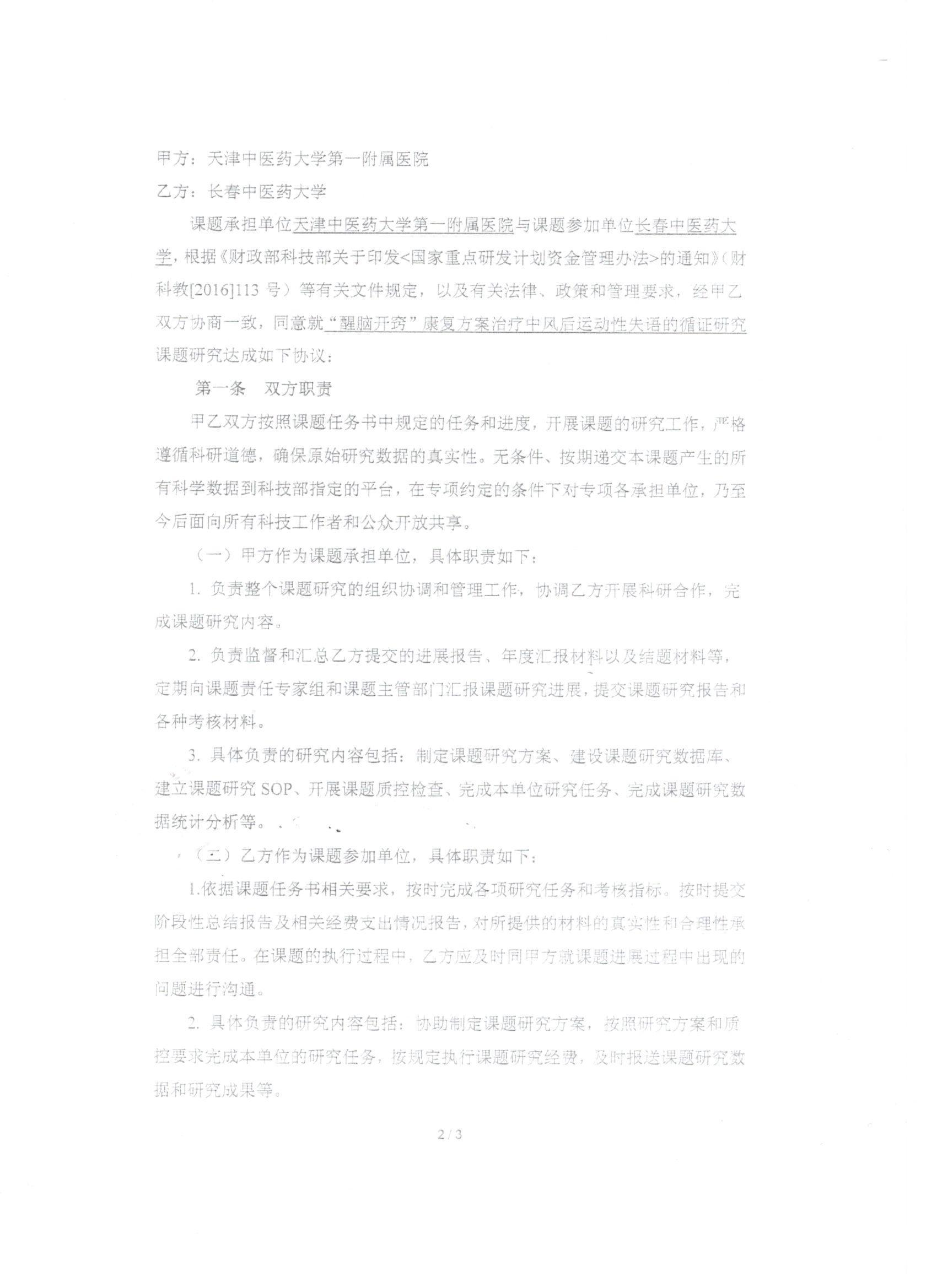


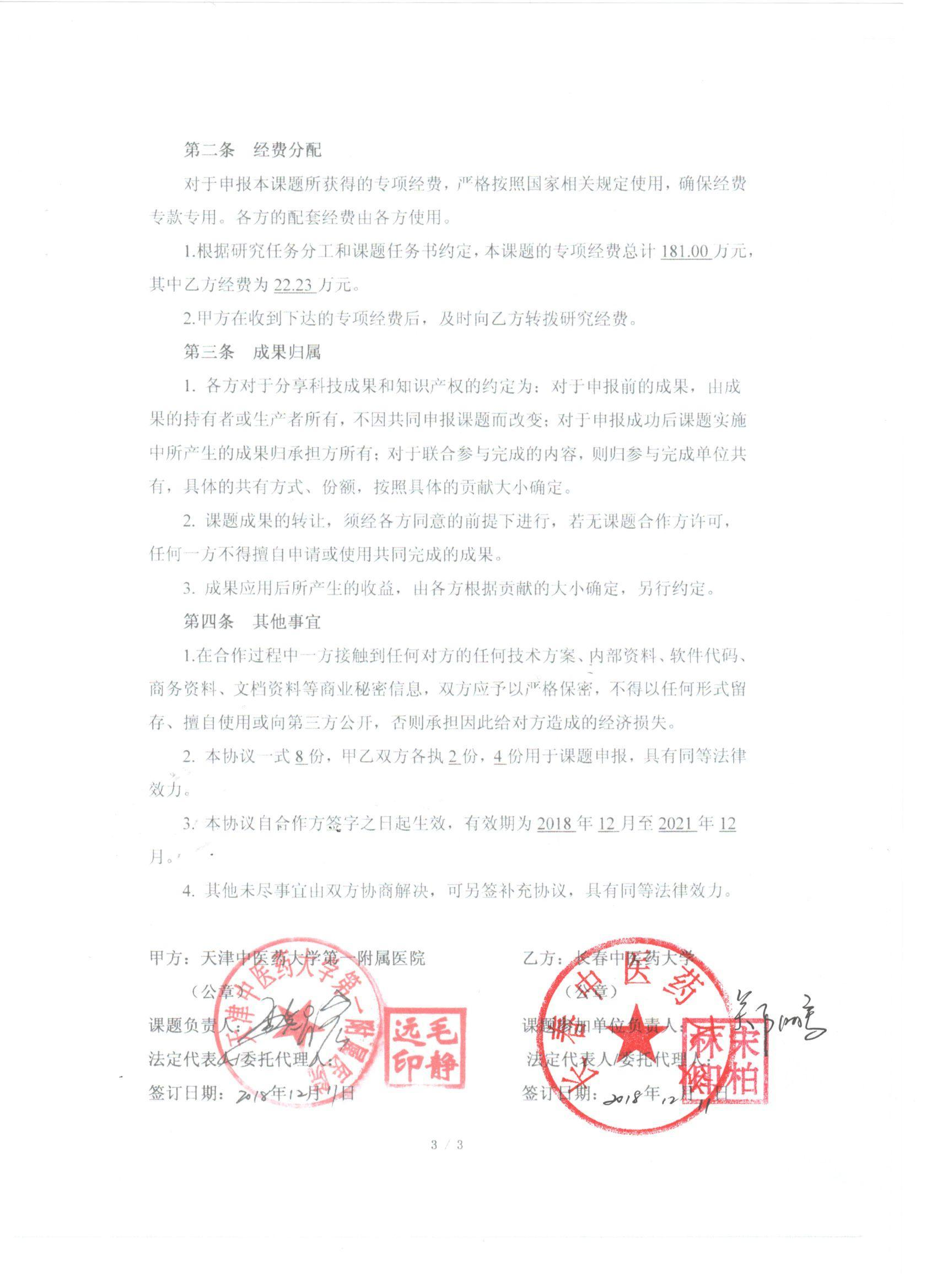


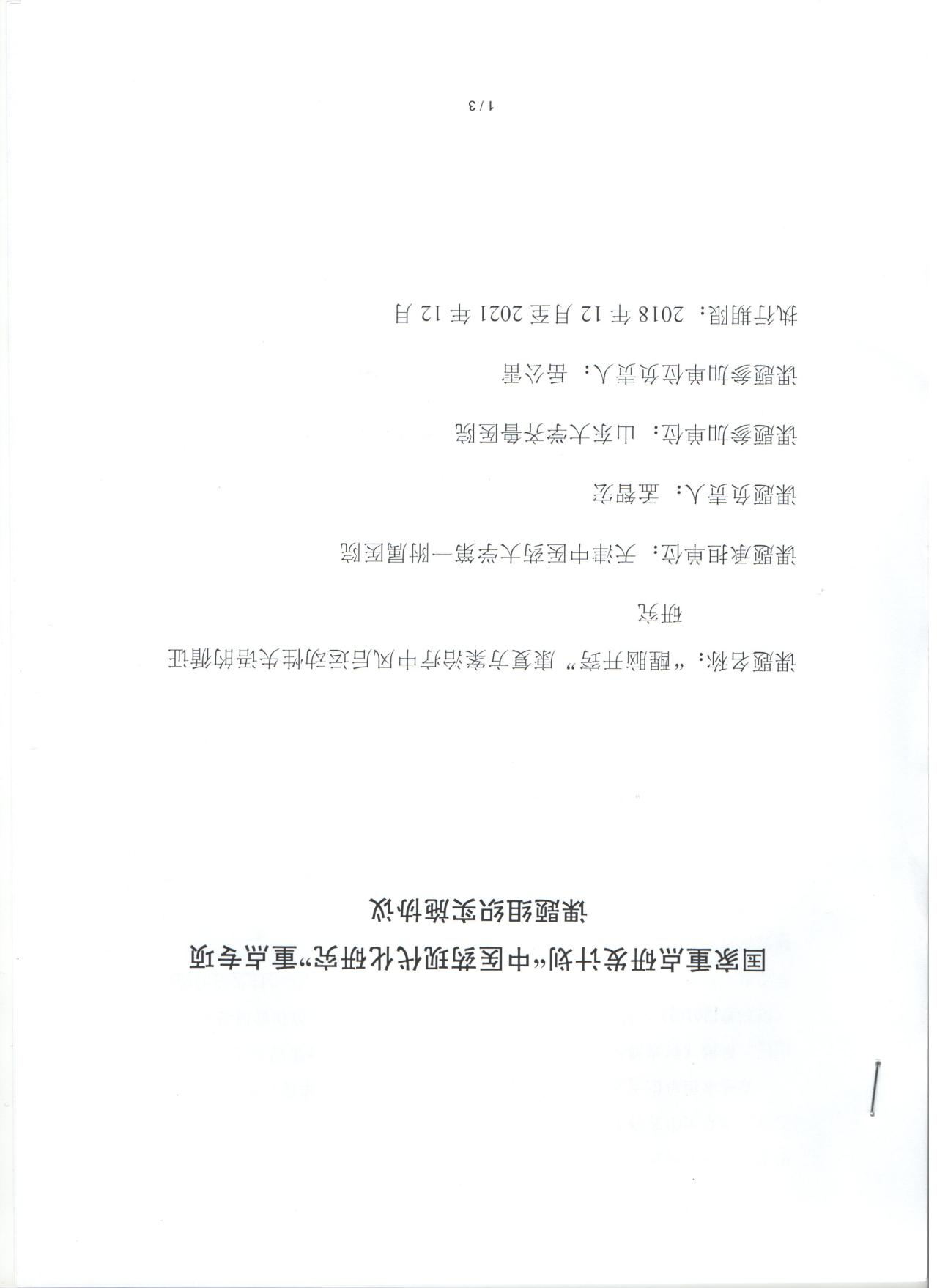


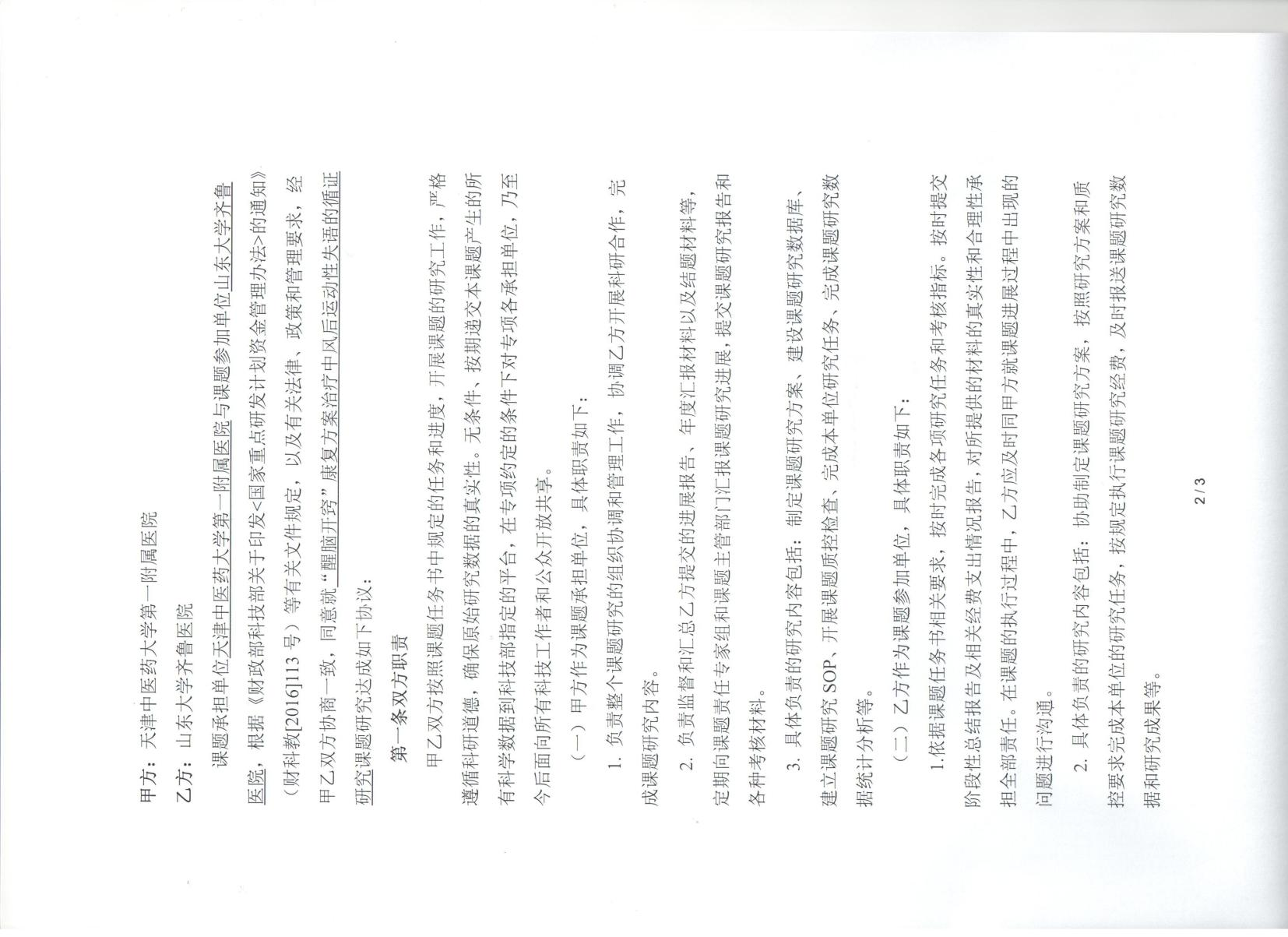


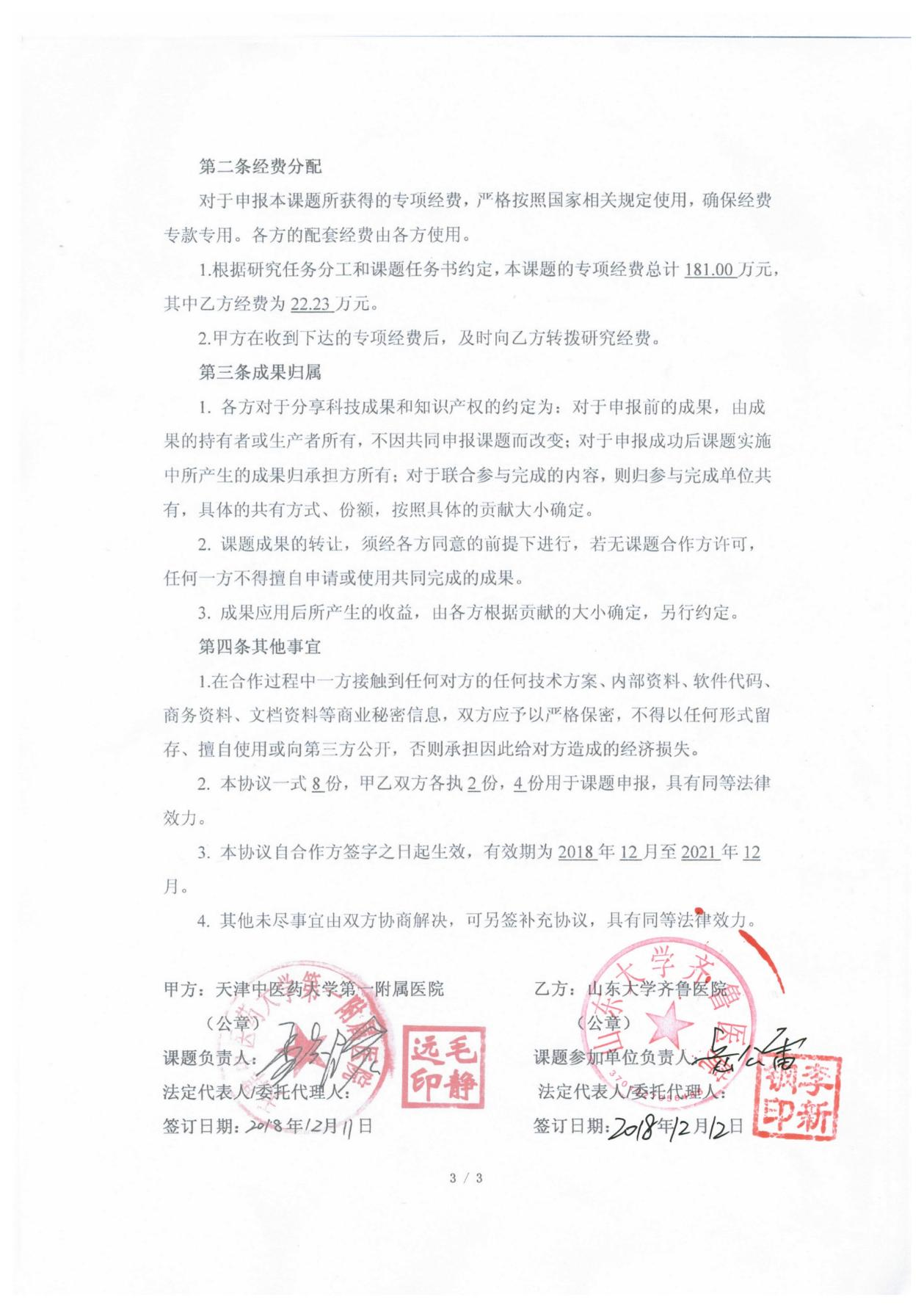

Supplement: Supplementary file 4 — Additional file 4. [file 13063_2022_6280_MOESM4_ESM.docx]
